# Dietary and Supplement-Based Complementary and Alternative Medicine Use in Pediatric Autism Spectrum Disorder

**DOI:** 10.3390/nu11081783

**Published:** 2019-08-01

**Authors:** Melanie S. Trudeau, Robyn F. Madden, Jill A. Parnell, W. Ben Gibbard, Jane Shearer

**Affiliations:** 1 Department of Kinesiology, University of Calgary, 2500 University Dr NW, Calgary, AB T2N 1N4, Canada; 2 Department of Health and Physical Education, Mount Royal University, Calgary, AB T3E 6K6, Canada; 3 Department of Pediatrics, Cumming School of Medicine, University of Calgary, Alberta Children’s Hospital, 28 Oki Drive NW, Calgary, AB T3B 6A8, Canada

**Keywords:** Autism spectrum disorder, dietary supplements, pediatric, physician communication

## Abstract

Previous literature has shown that complementary and alternative medicine (CAM) is steadily increasing in autism spectrum disorder (ASD). However, little data is currently available regarding its use, safety, and efficacy in children with ASD. Thus, the purpose of this study is to describe the use of supplement-based CAM therapies in children between the ages of 4 to 17 years with ASD. This population-based, cross-sectional study evaluated children with ASD regarding supplement use. A total of 210 participants were recruited from a variety of sources including educational and physical activity programs, and social media to complete a questionnaire. Primary caregivers provided information on current supplement based CAM use. Data evaluated the proportion of children that used supplement therapies, the types of supplements used, reasons for use, perceived safety, and demographic factors associated with use (e.g., income, parental education, severity of disorder). Seventy-five percent of children with ASD consumed supplements with multivitamins (77.8%), vitamin D (44.9%), omega 3 (42.5%), probiotics (36.5%), and magnesium (28.1%) as the most prevalent. Several supplements, such as adrenal cortex extract, where product safety has not yet been demonstrated, were also reported. A gluten free diet was the most common specialty diet followed amongst those with restrictions (14.8%). Health care professionals were the most frequent information source regarding supplements; however, 33% of parents reported not disclosing all their child’s supplements to their physician. In conclusion, the use of supplement therapies in children with ASD is endemic and highlights the need for further research concerning public health education surrounding safety and efficacy.

## 1. Introduction

Autism spectrum disorder (ASD) is a group of heterogeneous chronic neurodevelopmental disorders characterized by qualitative impairments in social interaction, communication, and repetitive stereotyped patterns of behavior [1]. The etiology of these conditions is thought to be multifactorial, involving genetic, prenatal, and postnatal factors [2]. The Centre for Disease Control (CDC) reports that 1 in 59 children are diagnosed with ASD, with boys 4 times more likely to be diagnosed than girls. As such, ASD is the fastest growing developmental disorder in the United States [3]. 

Treatment for ASD focuses on educational and behavioral interventions such as applied behavioral analysis [4]. Psychotropic drugs are commonly prescribed to treat core behavioral symptoms, decrease maladaptive behavior, and support learning and development [5]. In addition to conventional treatment options, some parents of children with ASD seek out complementary and alternative medicine (CAM) to treat symptoms. The National Centre for Complementary and Integrative Health defines CAM as “a diverse group of medical and health care systems, practices, and products that are not generally considered part of conventional Western medicine” [6]. Complementary approaches fall broadly into 3 categories: Natural products such as dietary supplements and special diets, mind and body practices, and other complementary health approaches [7].

Evidence regarding the use of CAM in the general pediatric population is limited. Studies in the United States have shown that the prevalence of pediatric CAM use in populations with illness or disease can range up to 76% [8]. However, these studies are limited in several ways. First, while many employ sound methodologies, they often provide differing definitions of what constitutes a CAM therapy. For example, in their review of 136 studies on alternative medicines, Surette et al. [9] found 39 studies that included vitamins, 13 studies that excluded vitamins, and 41 studies that made no mention of their inclusion or exclusion criteria. Further, many of the pediatric CAM studies are characterized by wide variation in study populations and size, prevalence measurements, and research methodologies, all of which hinder the formulation of evidence-based recommendations.

Though limited in number, some studies have examined CAM effects in ASD. Levy et al. [10] found that greater than 9% of children with ASD used potentially harmful CAM, such as chelation, antibiotics, or excessive amounts of vitamins. These findings are consistent with anecdotal evidence of dangerous products used to “cure” ASD. For example, in 2014, the supplementation market saw an explosion of *Miracle Mineral Solution,* a solution of sodium chlorite and hydrochloric acid (i.e., bleach) as a treatment option for ASD. The US Food and Drug Administration has issued several warnings about the product and the treatment has been linked to 1 death and several serious injuries; however, *Miracle Mineral Solution* is still widely available, with 1000 + followers on social media promoting its use [11]. 

From a public health perspective, supplement-based therapies and specialty diets, a subcategory of CAM, requires further evaluation. While many supplements such as melatonin, vitamins, gluten-casein-free diet, and omega 3 fatty acids may have few adverse effects, their safety and effectiveness in reducing ASD symptomology have not been reliably established [2,12,13]. Research estimates that up to 74% of children with ASD have been provided with CAM and that supplement-based therapies make up approximately 50% of CAM therapies used by this population [14].

Despite its popularity, disclosure of CAM use to physicians is often poor, with rates as low as 23% [15]. Concurrent use of CAM and prescription medications is widespread and poses a possible risk to patients who may be unaware of the potential for interactions [16]. Further, research has documented that knowledge of CAM use is important for health care professionals, as it provides insight into patient values and health beliefs. Importantly, considering patient values may assist in providing optimum care, especially in the context of supplements that pose a safety risk to patients [15]. Given the rates of concurrent use, in conjunction with lack of disclosure, there is a pressing need to assess pediatric CAM use and parental perceptions of these therapies.

As the prevalence of supplements and specialty diets are high and many are unsupported by research, a better understanding of the use of supplementation in pediatric ASD could help provide better integrative care by (1) informing the public and health care professionals about the prevalence and types of supplement therapies and specialty diets used in children with ASD; (2) assessing patient-physician communication and interactions surrounding supplement and specialty diet use; and (3) highlighting priorities for evidence-based clinical trials for supplements in ASD. Therefore, this study seeks to describe the use of supplement-based CAM therapies in children with ASD.

## 2. Materials and Methods 

### 2.1. Participants

This study was approved by the Conjoint Health Research Ethics Board at the University of Calgary (REB17-0970). Inclusion criteria for this population-based cross sectional study included (1) a physician-confirmed diagnosis of autism spectrum disorder (including previous diagnostic labels of Asperger’s syndrome, pervasive developmental disorder—not otherwise specified, childhood disintegrative disorder, or Rett syndrome) and (2) between the ages of 4 and 17.99 years inclusive. A sample size calculation (margin of error 8% and 95% confidence interval) revealed 150 participants were required [17]. Many of the children were cognitively and/or developmentally delayed; therefore, the parents/legal guardians served as a proxy for describing their child’s use of supplements, in order to maintain consistency between responses. The parents/legal guardians of children with ASD provided written informed consent and completed the questionnaire on their behalf.

### 2.2. Dietary Supplement Questionnaire

Data about supplement use with regards to ASD was collected via self-report online and paper form questionnaires. A validity and reliability tested supplement use questionnaire [18,19] was modified for children with ASD. Supplements were defined as a product that contains a vitamin, mineral, herb or botanical, amino acid, concentrate, metabolite, or other dietary ingredients intended to add further nutritional value to the diet. Supplements may be found in many forms such as tablets, capsules, soft gels, liquids, or powders. Examples include multivitamins, supplementary minerals, protein powders, energy drinks, meal replacements, etc. This definition was based on the definition provided by the National Centre for Complementary and Integrative Health [6]. Specialty diets such as the ketogenic diet, low-carb diet, and gluten free diet were also evaluated. The response format for the survey contained several closed-ended questions, short answer, and 5 item Likert scale questions. Several questions provided participants with answers to select from as well as short answer boxes to provide their answers. A pilot-test was conducted on a small sample of parents (*n* = 34) to ensure clarity of content. A sample of the questionnaire can be found in Supplementary Data File S1, Table S1.

### 2.3. Measures

The key outcomes were the demographic variables of the child (i.e., age, sex, ethnicity, medical characteristics) and parent (i.e., income, education level), and the types of therapies (i.e., gluten free diet, omega 3 fatty acids, probiotics) used. Secondary outcomes included reasons why parents have or have not used supplement therapies for their children, the information sources consulted by parents regarding therapies, and the proportion of parents who perceive the therapies used as being safe. In addition, parent perceived satisfaction with their child’s family physician or pediatrician, comfort level in discussing nutrition and supplements, number of supplements/dietary patterns disclosed to the physician (if applicable), and reasons why they might have chosen not to disclose supplements were quantified. 

### 2.4. Procedures

Children were recruited from clinics at the Alberta Children’s Hospital, Autism Calgary, and physical literacy programs around the city. Researchers also utilized schools that focus on inclusive and accessibility programs to recruit eligible participants. Recruitment posters were displayed throughout the facilities to describe the study, explain eligibility criteria, and provide contact information should parents of children with ASD want to participate. Researchers also contacted program organizers and asked permission to approach parents directly during recreation and instructional sessions and enroll them in the study. Many of these organizations promoted the study on their social media platforms, which provided an online consent form and a link to complete the survey.

### 2.5. Statistical Analysis

Data from the question ‘has your child previously taken or currently taking any dietary supplements’ was categorized as yes or no. Dietary supplement use data were categorized into groups based on sex, age (4 to 8 years, 9 to 13 years, and 14 to 17 years), and abilities. The age groups were based on dietary reference intake (DRI) values [20]. Ability groups were based on 4 ability categories (verbal, intellectual, social, and physical) in which parents ranked their child as “very weak/weak,” “neutral,” or “strong/very strong. Differences between sex, age, and ability groups were determined by a Fisher’s exact test. All analyses were performed using SPSS statistics version 25 (IBM Corporation, Armonk, NW, USA). 

## 3. Results

### 3.1. Participant Characteristics 

A total of 210 parents agreed to participate in the study on behalf of their child(ren) and completed the questionnaire. Descriptive characteristics and demographic characteristics of the participants are outlined in Table 1 and Table 2, respectively. 

### 3.2. Dietary Supplement Use 

A total of 167 parents (79.5%) indicated that their child had previously taken or was currently taking at least 1 supplement (males 77.1%; females 86.8%, *p* = 0.168). Eighty-three percent (83%) of parents reported thinking supplements were safe, 3.8% reported that they did not consider them safe, and 13.3% were undecided or perceived supplements as safe under certain conditions (i.e., evidence-based, supervised by a health care professional, etc.). There were no statistical differences between sexes in perceived safety of dietary supplement use (*p* = 234). 

**Figure 1:**
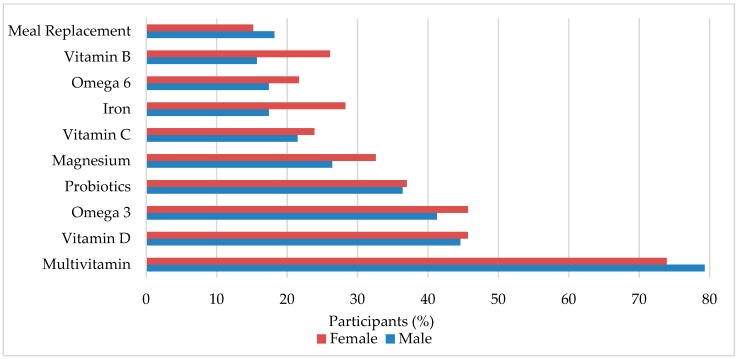
Dietary supplements commonly used in pediatric autism spectrum disorder (ASD). Male and female is percent within sex.

The top 10 previous and current pediatric supplement use is summarized in Figure 1. A more extensive list of the supplements listed by the questionnaire can be found in Supplementary Data, Table S2. The 5 most common supplements were multivitamins (77.8%), vitamin D (44.9%), omega 3 (42.5%), probiotics (36.5%), and magnesium (28.1%). Other supplements such as alpha lipoic acid, sodium butyrate, N-Acetyl Cysteine, 5HTP, fluoride, methylfolate, adrenal cortex extract, selenium, milk thistle, liposomal curcumin, cannabidiol, and melatonin were also mentioned. Differences between sexes were found in calcium (male = 9.1%, female = 21.7%, *p* = 0.037) and vitamin K (male = 1.7%, female = 10.9%, *p* = 0.018). When analyzed by age group, significant differences were found in calcium (*p* = 0.049), vitamin B (*p* = 0.010), and energy drinks (*p* = 0.027). The percentage of children who have/are taking more than 1 supplement was 88.6%. In addition, a Pearson’s correlation determined that there was no relationship between number of supplements used and years since diagnosis (*p* = 240). The average number of supplements per child was 4.49 and the mean year of diagnosis was 2013. 

Of the 167 parents who indicated their child has or is currently taking supplements, 126 rated the perceived degree of change on their child’s overall well-being between neutral and positive (69.1 ± 15.9) on a scale of 1 (negative impact) to 100 (positive impact). The most common supplements cited to have the largest impact on the child’s health were melatonin, multivitamins, omega fatty acids, and magnesium.

### 3.3. Dietary Supplement Reasons for Use

Parental reasons for providing their children with supplements are outlined in Table 3. The top 3 reasons for consuming supplements were to enhance the child’s diet, promote immune system function, and increase quality/duration of sleep. Improvement in gut health was listed 7 times under the “other” section. Parents also indicated several reasons for omitting supplements including: Inadequate knowledge/information (*n* = 14), too expensive (*n* = 8), and may be considered harmful (*n* = 5). Six parents indicated that supplements were not necessary, as their child eats a balanced diet.

### 3.4. Special Diets and Information Sources

Current diet information is summarized in Table 4. The top 4 diets followed in our sample included no restrictions, gluten free, high carbohydrate, and lactose free, with a significant difference between sex in diets with no restrictions (male = 72.0%, female = 54.7%, *p* = 0.027), gluten free (male = 5.1%, female = 24.5%, *p* < 0.001), and lactose free (male = 4.5%, female = 17.0%, *p* = 0.006). Other diets such as the paleo diet, nut-free, dye-free, and low sugar diets were also mentioned. In addition, 6 parents also stated that their children were picky eaters and had “very limited diets.” 

**Figure 2:**
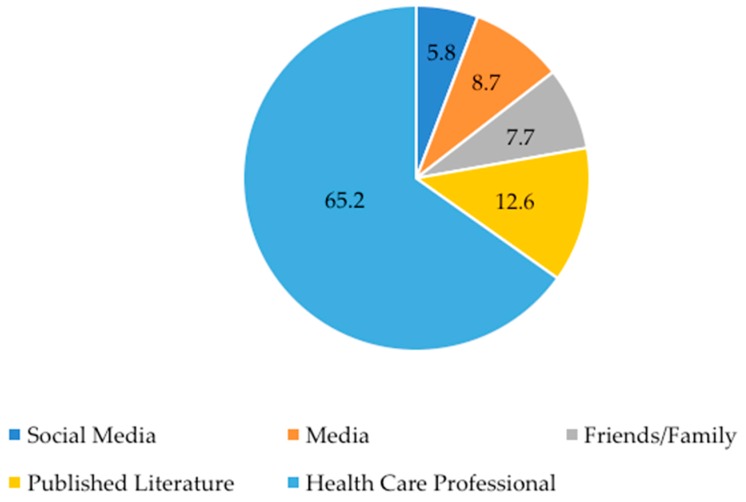
Primary information sources about dietary supplements. Data is presented as % of respondents. Participants could only choose one source or answer in the “other” section. One participant indicated in the “other” section that they use a combination of social media and health care professionals, so a “yes” was put into each category.

The primary information sources regarding dietary supplements used by parents are shown in Figure 2. Sixty-five percent (65%) of parents indicated that health care professionals (e.g., physician, nurse, nutritionist) were their primary source of information regarding dietary supplements. Published literature and media (e.g., news, magazines, journals) were listed as the second and third most popular sources of information. Social media was the least utilized source, with only 5.8% of parents indicating use. Two participants did not indicate a primary information source and two other participants indicated “knowledge over the years,” and “myself (pharmacist)” in the “other” section.

### 3.5. Physician Communication

Nearly all families (98.1%) indicated that they have a family doctor and/or a pediatrician. Eleven percent (11%) of families (*n* = 21) rated their overall level of satisfaction with the primary care physician as not satisfied or partially unsatisfied on a scale of 1 (not satisfied) to 5 (very satisfied), whereas 87% of parents (*n* = 169) rated their level of comfort in discussing supplements with physicians as comfortable or very comfortable. A large majority (72.4%; *n* = 152) had never met with a dietician. The frequency of disclosure of supplement use to a physician is summarized in Table 5. Results revealed that 33.5% of parents did not disclose all supplements to their physician. Several reasons were reported for omitting disclosure of supplementation use to primary care physicians including perceived “physician lack of knowledge”, “no benefit”, “too time consuming”, and “scared of judgment”.

## 4. Discussion

The use of complementary and alternative medicine (CAM) is increasing among children and is common in those with chronic illness or disorders, such as ASD [13]. Critically, however, a complete profile of dietary supplement use in children with ASD is lacking; making it difficult to develop educational strategies. The present study is impactful, as it provides a fully powered assessment of supplement-based CAM therapies in children with ASD.

### 4.1. Dietary Supplement Patterns and Special Diet Use 

In this study, 75.9% of the children sampled have consumed dietary supplements, supporting the upper end of the prevalence range of supplement use in pediatric ASD cited in previous literature [14]. As the most recent studies evaluating supplement use in children with ASD were conducted several years ago, it is possible that the high prevalence reported here reflects a continued increase in supplement use. Additionally, this study utilized a broad definition of supplements when compared to other studies, therefore, it is likely we captured a wider variety of supplements used by children with autism. Nevertheless, the reported high rates of dietary and supplement use in this study would indicate these products continue to be of interest as a complementary approach to standard treatment of care. 

Multivitamins, vitamin D, omega 3 fatty acids, probiotics, and magnesium were the most common supplement therapies used. Research regarding the efficacy of these supplements in ASD populations requires further evaluation [21]. Multivitamins, for example, are considered a popular CAM therapy in ASD [22]. The rationale for this treatment is based on the frequently observed dietary deficiency of vitamins and micronutrients in children with ASD. Children with ASD are often deficient in calcium, vitamin D, vitamin K, vitamin A, vitamin E, zinc, vitamin B6, and tetrahydrobiopterin [21]. These deficiencies could be the result of food selectivity or altered gastrointestinal absorption. Adams et al. [23] conducted a double-blind randomized control trial to examine the effect of a common commercial vitamin supplement on observed improvements in parent-rated pre and post autism symptomatology. They found significant improvements in hyperactivity, tantrumming, overall, and receptive language, suggesting it as a reasonable adjunct therapy for children with ASD. However, no other study has evaluated or been able to replicate the effectiveness or safety of this biological therapy. Similarly, preliminary studies on probiotics have shown improvement in core symptomatology in ASD [21], but they have been minimally replicated and several other studies have denounced their effects. It is also possible that there are responders and non-responders to individual treatments, further complicating interpretation. 

Recently, vitamin D has been proposed as a potential treatment for ASD [24]. In 2015, an open trial demonstrated significant improvements in autism rating scales following 3 month vitamin D3 supplementation [25]. However, this small sample study has been the only experimental study to demonstrate the potential efficacy of vitamin D in children with ASD, highlighting the need for more wide-scale studies to critically validate the efficacy of vitamin D before drawing any definite conclusions. 

Collectively, these supplements require more systematic and rigorous research. As a result, there is little evidence to support the use of any nutritional supplement or dietary therapy for children with ASD [26]. Furthermore, some of the other supplements reported in this study confirm anecdotal reports regarding the consumption of dangerous biological therapies. The Food and Drug Administration has issued several health warnings about adrenal cortex extract, for example, and has deemed it “unsafe and ineffective for labeled indications for human use” [27,28,29]. Evidently, there are gaps in the transmission of scholarly literature to quality educational materials for families, as many children continue to consume dietary supplements that are unsupported by research. In addition, several parents indicated that they were undecided about the safety of supplements and listed inadequate knowledge about supplements as the number one factor barring use.

Research evaluating specialty diets (e.g., gluten free casein free, lactose free, etc.) shows similar ambiguity. More specifically, there are few studies that demonstrate conclusive results in the gluten free and casein (or lactose) free diets reported in this study [30]. Many are small in size and lack strict dietary controls, both common problems in conducting dietary research in children, which limits the ability of researchers to drawn firm conclusions. Consequently, many studies regarding specialty diets point to the need for further research and illustrate how clinicians often find themselves unable to offer the most up-to-date and scientifically credible information to their patients. Of note, the ketogenic diet has emerged as a leader in specialty diets for ASD in the past several years and has offered promising, though preliminary, results in both animal and human studies [31,32,33]. Seven participants reported utilizing this diet. 

### 4.2. Physician-Patient Communication 

Sixty-five percent (65%) of parents disclosed that their primary source of information regarding supplements were health care providers. In addition, 72% of families indicated that they had never met a dietician, signifying that many are relying on their physician for quality information regarding supplements and special diets. However, 33% admitted to not disclosing all supplements to their physician due to perceived physician lack of knowledge, no apparent benefit, the time commitment, and fear of judgment. Alarmingly, as 36.7% reported taking prescribed medication, a lack of disclosure may pose a risk to patients who may be unaware of the potential for interactions with concurrent CAM use. An open, patient-centered, non-judgmental approach is recommended for physicians when discussing supplement therapies [34]. This study highlights that patients would like to receive information about CAM from their conventional health care team, underscoring the importance of clinician knowledge about CAM and emerging research findings.

### 4.3. Limitations

There are a couple areas to consider when examining the limitations of this study. While the study provides novel information about the use of supplement therapies and special diet use in children with ASD, the study is limited as the majority of its participants are from Canada, therefore, may not be generalizable to other geographic regions. Further, as this is a descriptive study, it does not provide causal information regarding the effect of individual supplement therapies (e.g., omega 3 fatty acids causing relief of gastroenteritis symptoms). Finally, social desirability, a common bias where respondents answer in a way viewed favorably by others, may have influenced the data. 

## 5. Conclusions

Supplement use continues to be a prevalent form of CAM used in ASD. While a variety of supplements and dietary interventions are utilized, the scientific consensus remains that there is currently little evidence to support the use of any nutritional supplement or dietary therapy for children with ASD. Future investigation into the effects of individual supplements on physiological and psychological functioning to determine optimal supplementation strategies in ASD is required. 

## Figures and Tables

**Table 1:** Descriptive characteristics.

Descriptive Characteristics	All	Males	Females
Participants	210	157 (74.8%)	53 (25.2%)
Age, years	9.2 (3.6)	9.0 (3.6)	9.5 (3.7)
Year of Diagnosis			
2003–2007	16 (7.8%)	14 (6.8%)	2 (1.0%)
2008–2012	42 (20.5%)	28 (13.7%)	14 (6.8%)
2013–2018	147 (71.7%)	113 (55.1%)	34 (16.6%)

Participants and year of diagnosis are listed as a count (percentage of total); age is listed as a mean (standard deviation).

**Table 2:** Demographic characteristics.

Characteristic	All *n* (%)	Males *n* (%)	Females *n* (%)
**Ethnicity**			
Caucasian	170 (81.3)	124 (59.3)	46 (22.0)
Asian/Pacific Islander	13 (6.2)	11 (5.3)	2 (1.0)
Hispanic or Latino	2 (1.0)	2 (1.0)	0 (0.0)
Black or African American	5 (2.4)	2 (1.0)	3 (1.4)
First Nations or Metis or Inuit	6 (2.9)	5 (2.4)	1 (0.5)
Multiracial	13 (6.2)	12 (5.7)	1 (0.5)
**Household Income**			
>$20,000	10 (4.8)	7 (3.3)	3 (1.4)
$20,000–40,000	23 (11.0)	18 (8.6)	5 (2.4)
$40,000–60,000	23 (11.0)	17 (8.1)	6 (2.9)
$60,000–80,000	33 (15.7)	25 (11.9)	8 (3.8)
$80,000–$100,000	35 (16.7)	23 (11.0)	12 (5.7)
$100,000+	75 (35.7)	60 (28.6)	15 (7.1)
Not Applicable	11 (5.2)	7 (3.3)	4 (1.9)
**Parent Level of Education**			
High School Diploma	17 (8.2)	13 (6.3)	4 (1.9)
Trade School Diploma	4 (1.9)	3 (1.4)	1 (0.5)
Some College	17 (8.2)	13 (6.3)	4 (1.9)
College Diploma	51 (24.5)	41 (19.7)	10 (4.8)
Some University	19 (9.1)	17 (8.2)	2 (1.0)
University Degree	52 (25.0)	40 (19.2)	12 (5.8)
Master’s Degree	30 (14.4)	21 (10.1)	9 (4.3)
Professional Degree	11 (5.3)	5 (2.4)	6 (2.9)
Ph.D.	7 (3.4)	3 (1.4)	4 (1.9)

The category “Multiracial” was created as a result of multiple parents indicating this option in the “other” category to reflect the demographic of our sample. Data is presented as a count (percentage of total).

**Table 3:** Reasons for and against dietary supplement use.

	All *n* (%)	Males *n* (%)	Females *n* (%)	*p*
**Reasons for Use**				
Enhance diet	127 (76.0)	94 (56.3)	33 (19.8)	0.424
Promote immune system function	88 (52.7)	66 (39.5)	22 (13.2)	0.490
Increase quality/duration of sleep	76 (45.5)	53 (31.7)	23 (13.8)	0.602
Improve cognitive ability	54 (32.3)	34 (20.4)	20 (12.0)	0.066
Decrease repetitive or restrictive behavior	21 (12.6)	16 (9.6)	5 (3.0)	0.798
Promote sociability	18 (10.8)	12 (7.2)	6 (3.6)	0.581
Increase interactions with others	13 (7.8)	8 (4.8)	5 (3.0)	0.349
Enhance motor skills	12 (7.2)	9 (5.4)	3 (1.8)	1.000
**Reasons Against Use**				
Inadequate knowledge/information	14 (32.6)	12 (27.9)	2 (4.7)	1.000
Too expensive	8 (18.6)	7 (16.3)	1 (2.3)	1.000
May be considered harmful	5 (11.6)	5 (11.6)	0 (0.0)	0.574
Based on suggestion from close family/friends	4 (9.3)	4 (9.3)	0 (0.0)	1.000
Read in scholarly article	4 (9.3)	4 (9.3)	0 (0.0)	1.000

Reasons for use are listed for the parents who indicated that their child had taken supplements (*n* = 167). Reasons against use are listed for the parents who indicated that their child had not taken supplements (*n* = 43). Data is presented as a count (percentage of total).

**Table 4:** Special diet use in pediatric autism.

	All *n* (%)	Males *n* (%)	Females *n* (%)	*p*
No restrictions	142 (67.6)	113 (72.0)	29 (54.7)	**0.027**
Gluten free	21 (10.0)	8 (5.1)	13 (24.5)	**<0.001**
High carb	17 (8.1)	14 (8.9)	3 (5.7)	0.570
Lactose free	16 (7.6)	7 (4.5)	9 (17.0)	**0.006**
Casein free	12 (5.7)	8 (5.1)	4 (7.5)	0.734
High protein	8 (3.8)	4 (2.5)	4 (7.5)	0.206
Ketogenic diet (i.e., high fat, low card)	7 (3.3)	6 (3.8)	1 (1.9)	0.682
Vegetarian	4 (1.9)	2 (1.3)	2 (3.8)	0.574
Vegan	0 (0.0)	0 (0.0)	0 (0.0)	n/a

Diet data is presented as a count (percentage within sex) who follow each diet. Differences between sex were determined using a Fisher’s exact test. *p* < 0.05 was considered significant. Significant differences are bolded. n/a, not applicable.

**Table 5:** Disclosure of supplement use to physician.

Disclosure of Number of Supplements (%)	Frequency *n* (%)
None (0)	11 (6.6)
Some (1–49)	14 (8.4)
Half (50)	8 (4.8)
Most (51–99)	23 (13.8)
All (100)	111 (66.5)

Frequency data is presented as a count (percentage of total).

## Supplementary Materials

The following are available online at https://www.mdpi.com/2072-6643/11/8/1783/s1, File S1: Dietary Patterns and Supplement Use in Pediatric Autism, Table S1. All Supplement Use in Children with ASD, Table S2. Supplements listed in the “Other” Category by Parents.
