# Supplementary material for: Dietary and Supplement-Based Complementary and Alternative Medicine Use in Pediatric Autism Spectrum Disorder
Source: Nutrients. 2019 Aug 1;11(8):1783. doi: 10.3390/nu11081783 (PMC6724073; doi:10.3390/nu11081783)
Supplement: Supplementary file 1 [file nutrients-11-01783-s001.pdf]

Table S1. All Supplement Use in Children with ASD

Supplement	All n(%)	Male n(%)	Female n(%)	*p*
Multivitamin	130 (77.8)	96 (79.3)	34 (73.9)	.532
Calcium	21 (12.6)	11 (9.1)	10 (21.7)	**.037**
Magnesium	47 (28.1)	32 (26.4)	15 (32.6)	.446
Vitamin A	12 (7.2)	8 (6.6)	4 (8.7)	.738
Vitamin D	75 (44.9)	54 (44.6)	21 (45.7)	1.000
Vitamin C	37 (22.2)	26 (21.5)	11 (23.9)	.835
Vitamin B	31 (18.6)	19 (15.7)	12 (26.1)	.180
Vitamin K	7 (4.2)	2 (1.7)	5 (10.9)	**.018**
Iron	34 (20.4)	21 (17.4)	13 (28.3)	.135
Zinc	22 (13.2)	14 (11.6)	8 (17.4)	.317
Folic Acid	7 (4.2)	5 (4.1)	2 (4.3)	1.000
Antioxidants (e.g. vitamin E)	16 (9.6)	10 (8.3)	6 (13.0)	.213
Amino Acids	8 (4.8)	5 (4.1)	3 (6.5)	.686
Creatine	6 (3.6)	5 (4.1)	1 (2.2)	1.000
Protein Powders	18 (10.8)	12 (9.9)	6 (13.0)	.581
Omega 3	71 (42.5)	50 (41.3)	21 (45.7)	.726
Omega 6	31 (18.6)	21 (17.4)	10 (21.7)	.511
Fibre	28 (16.8)	16 (13.2)	12 (26.1)	.063
Vitaminized Water	4 (2.4)	3 (2.5)	1 (2.2)	1.000
Energy Drinks (e.g. Redbull)	2 (1.2)	1 (0.8)	1 (2.2)	.476
Probiotics	61 (36.5)	44 (36.4)	17 (37.0)	1.000
Meal Replacement (e.g. Ensure)	29 (17.4)	22 (18.2)	7 (15.2)	.820

Intakes are presented as the number of children (%) who have or are currently consuming each dietary supplement. Male and female are presented as percent within gender. Differences between genders were determined using a Fisher's Exact test. $p < 0.05$ was considered significant. Significant difference are bolded.

Table S2. Supplements listed in the "Other" Category by Parents

Supplement	All n(%)	Male n(%)	Female n(%)	*p*
Melatonin	27 (16.2)	19 (15.7)	8 (17.4)	.636
Digestive Enzymes	2 (1.2)	1 (0.8)	1 (2.2)	n/a
Sodium Butyrate	1 (0.6)	1 (0.8)	0 (0.0)	n/a
Thiamine	1 (0.6)	1 (0.8)	0 (0.0)	n/a
Feverfew	1 (0.6)	1 (0.8)	0 (0.0)	n/a
Fluoride	1 (0.6)	1 (0.8)	0 (0.0)	n/a
N-Acetyl Cysteine	1 (0.6)	0 (0.0)	1 (2.2)	n/a
5HTP	1 (0.6)	0 (0.0)	1 (2.2)	n/a
Alpha Lipoic Acid	1 (0.6)	1 (0.8)	0 (0.0)	n/a
Inulin	1 (0.6)	1 (0.8)	0 (0.0)	n/a
Olive Oil	2 (1.2)	1 (0.8)	1 (2.2)	n/a
L-Methyl Folate	1 (0.6)	1 (0.8)	0 (0.0)	n/a
Adrenal Cortex	1 (0.6)	1 (0.8)	0 (0.0)	n/a
Selenium	1 (0.6)	1 (0.8)	0 (0.0)	n/a
Milk Thistle	1 (0.6)	1 (0.8)	0 (0.0)	n/a
Liposomal Curcumin	1 (0.6)	1 (0.8)	0 (0.0)	n/a
Cannabidiol	1 (0.6)	1 (0.8)	0 (0.0)	n/a
Calorie Powder	1 (0.6)	0 (0.0)	1 (2.2)	n/a
Polyethylene Glycol 3350	1 (0.6)	1 (0.8)	0 (0.0)	n/a
Elderberry	1 (0.6)	1 (0.8)	0 (0.0)	n/a
Fermented Foods	1 (0.6)	1 (0.8)	0 (0.0)	n/a
Super Sleep	1 (0.6)	1 (0.8)	0 (0.0)	n/a
RestoraLAX	1 (0.6)	0 (0.0)	1 (2.2)	n/a
Spearmint Leaf Tea	1 (0.6)	0 (0.0)	1 (2.2)	n/a

Intakes are presented as the number of children (%) who have or are currently consuming each dietary supplement. Male and female are presented as percent within gender. No differences between genders were determined.
